# Associations between depressive complaints and indicators of labour participation among older Dutch employees: a prospective cohort study

**DOI:** 10.1007/s00420-020-01584-9

**Published:** 2020-10-21

**Authors:** Jacqueline G. M. Jennen, N. W. H. Jansen, L. G. P. M. van Amelsvoort, J. J. M. Slangen, I. J. Kant

**Affiliations:** Department of Epidemiology, School for Public Health and Primary Care (CAPHRI), Maastricht University, P. O. Box 616, 6200 MD Maastricht, The Netherlands

**Keywords:** Older workers, Depressive complaints, Labour participation, Longitudinal, Work context

## Abstract

**Purpose:**

European policy measures have led to an increased net labour participation of older employees. Yet, via different routes (for instance disability schemes) employees still often leave the labour market early. Mental health may be an important factor hindering labour participation. Aims of this study are twofold: first, to examine the relationship between mental health—particularly depressive complaints—and indicators of labour participation among older employees over a 2-year follow-up period and second, to explore the impact of different work contexts when studying this relation.

**Methods:**

A subsample of older employees (aged > 45 years; *n* = 1253) from the Maastricht Cohort Study was studied. Depressive complaints were assessed using the Hospital Anxiety and Depression scale. Logistic and Cox regression analyses covered 2 years of follow-up and were also stratified for relevant work-related factors.

**Results:**

Employees with mild depressive complaints showed statistically significantly higher risks for poor mental workability (HR 2.60, 95% CI 1.14–5.92) and high psychological disengagement levels (HR 2.35, 95% CI 1.21–4.57) over time compared to employees without depressive complaints. Within various work contexts, for instance in which employees perform physically demanding work or have high psychological job demands, significantly stronger associations were found between depressive complaints and poor mental workability over time.

**Conclusions:**

This study shows strong longitudinal associations between depressive complaints and indicators of labour participation, also within different work contexts over time. Results provide valuable input for developing preventive measure aiming to enhance sustainable labour participation of older employees.

## Introduction

In many developed Western countries, there is a need to increase labour market participation due to amongst others longevity and several policies have already been developed and implemented (European Commission 2014). The Europe 2020 strategy targets at increasing the working population’s employment rate to at least 75 per cent (European Commission 2014). Besides policies at the EU level, many national policy measures focus on increasing labour participation of older employees. Employees from 45 years onwards are defined as older employees by the WHO (Ilmarinen 2005). For instance, The Netherlands implemented policy measures such as increasing the legal retirement age and abolishing early retirement schemes (Doekhie et al. 2014; Ybema et al. 2009). These policy measures have effectively increased the net labour participation of older Dutch employees over a 10-year time period (2008–2017). A major increase was specifically noticeable for employees aged 55–65 years (Statistics Netherlands 2017a,^2018^): the net labour participation of men increased from 61.7 to 77.4% and for women from 39.6 to 59.2% in this period (Statistics Netherlands 2018).

Notwithstanding this increased labour participation, it is important to note that older employees still leave the labour market before the actual retirement age. For instance, in the Netherlands, this occurs via different routes such as unemployment or disability schemes. There is an increasing proportion of employees receiving work disability benefits according to the Work and Income by Work Capacity Act (Berendsen 2017). This proportion increased from 5.3 to 8.2% over 4 years (2012–2016) within the 60 plus age group (Berendsen 2017). Furthermore, although the net labour participation of Dutch employees is among the highest in Europe (Statistics Netherlands 2017b) the amount of working hours per week is among the least (Statistics Netherlands 2017b). The proportion of part-time employees in the EU is by far the highest in The Netherlands with nearly 47% (Eurostat 2018). For older male and female employees aged 60–64 the amount of working hours per week is respectively 24.4 and 22.2 h/week (European Commission 2016). This may suggest that older employees might adjust their work hours to be able to continue labour participation (Damman et al. 2013).

With ageing, the risk of having (one or more) chronic condition(s) substantially increases (Doekhie et al. 2014; Sociaal-Economische Raad 2016; Ybema et al. 2009). Additionally, due to declining health, the risk of work incapacity/disability increases (Berendsen 2017). Chronic conditions are considered as an important factor which could hinder older employees’ ability to continue labour participation until their retirement age. This raises the question if and under which conditions older workers with a chronic condition are able to continue to participate in the labour market.

Chronic conditions are not unambiguously to define. In general, mental and somatic chronic conditions are distinguished (Sociaal-Economische Raad 2016). Amongst mental chronic conditions, depressive complaints comprise the majority of mental health complaints. Depressive complaints comprise a depressed mood or relatively mild to moderate symptoms of depression. Moreover, depressive complaints might act as a precursor for (clinical) depression according to DSM criteria (Nuijen et al. 2018; Lexis et al. 2009). Depressive complaints contribute to an increase in societal costs and healthcare use (Kant et al. 2008; Lexis et al. 2009). In the general Dutch population the prevalence rate of depressive complaints is high and is expected to keep increasing. In 2017, respectively 8.3 and 15.1% of men and women had depressive complaints (Nielen et al. 2017; World Health Organization 2018). Depressive complaints are also highly prevalent among the working population (Harvey et al. 2009; Lexis et al. 2012). The first goal of this study is therefore to gain more insight into the impact depressive complaints might have on labour participation of older employees.

When studying the potential association between depressive complaints and labour participation among older employees, it is important to consider that labour participation is a broad concept which entails various outcome measures or indicators. Often studies considered one single indicator to comprise the concept labour participation such as retirement intentions or disability pension (Bültmann et al. 2008; Lahelma et al. 2015). To complement the concept labour participation, it is valuable to consider both objective (employment status) and subjective indicators representing labour participation. Therefore, this study will consider both these objective (such as employment status) and subjective indicators (such as retirement intentions, psychological disengagement, and general, mental and physical work ability) to complement the concept labour participation.

In this respect, the concept work ability is relevant since it concerns a (in)balance between work demands and an employees’ individual resources to meet these demands (Boschman et al. 2017; Ilmarinen et al. 1997). Good work ability can support employment whereas poor work ability might increase the risk of early retirement (Ilmarinen 2008). Second, retirement intentions can be relevant as these might affect the continuation of labour participation (European Commission 2012). Additionally, motivation can be conceptualized by psychological (dis)engagement levels since older employees often gradually disengage from work by reducing work investments, activities and motivation a couple of years before the actual retirement age (Damman et al. 2013).

The Conservation of Resources (COR) model is used as a theoretical framework (Hobfoll 1989) throughout this study to understand and explain the association between depressive complaints and indicators of labour participation. The COR theory offered fundamental insights that have guided research on amongst others coping with chronic illness (Holmgreen et al. 2017). The main proposition of this theoretical framework is that individuals strive to obtain, foster, and protect resources. Resources are things individuals centrally value such as health, self-esteem, or time (Grandey and Cropanzano 1999; Hobfoll 1989, 2018). Stress is a reaction to an environment in which there is an actual or perceived loss in resources (Grandey and Cropanzano 1999; Hobfoll 1989). With ageing there is an inevitable loss of resources which requests a rearrangement of available resources to compensate for failing ones (Hobfoll 2018). In our study, older employees with depressive complaints (may) have a loss in resources due to impaired health and ageing. This might cause stress and could affect an employees’ ability to cope with their (mental) chronic conditions while being involved in labour participation resulting in withdrawal or disengagement (Hobfoll 2018).

Most empirical studies so far have focused on the impact of depressive complaints on one single and particular outcome such as sickness absence or work disability (Andrea et al. 2004; de Graaf et al. 2012; Lexis et al. 2009; Bültmann et al. 2008; Lahelma et al. 2015; Mykletun et al. 2006). Moreover, most studies assessed specific defined occupational groups (Chevalier et al. 1996; Gommans et al. 2017; Lahelma et al. 2015; Stansfeld et al. 2013). Though many studies are prospective cohort studies with significant long follow-up periods (Bonde 2008), these studies were performed in different European countries (Bültmann et al. 2008; Mykletun et al. 2006). Due to differences in social insurance systems generalizations to other countries/settings may be hindered. Furthermore, research specifically into older employees with depressive complaints is lacking. Moreover, longitudinal studies to date often focused on (major) clinical depression (Kaila-Kangas et al. 2014; Knudsen et al. 2010; Mykletun et al. 2006) or common mental disorders (Knudsen et al. 2010; Lahelma et al. 2015) and did not specifically distinguish depressive complaints as exposure of interest. Focusing on depressive complaints instead of clinical depression in the work context is of particular interest since employees with a clinical depression might already left the labour market whereas employees with depressive complaints are often still actively involved in the labour market. Furthermore, it is valuable to study depressive complaints both continuously as well as categorically. Studying different categories of severity might explain stagnation or transition to a more severe category of depressive complaints over time (Nuijen et al. 2018). Conceptualizing depressive complaints on a continuum ranging from no/mild depressive complaints at one end of the spectrum to severe complaints at the other end (Cuijpers et al. 2004; Geiselman and Bauer 2000; Hjarsbech et al. 2011) encompasses the total range of complaints preceding the diagnosis of clinical depression (Hjarsbech et al. 2011; Lexis et al. 2009). Moreover, it is valuable to consider the presence of other mental/physical chronic condition(s) since this may lead to a less favorable course of depressive complaints (Nuijen et al. 2018) and consequently also affect labour participation.

The understanding of the association between depressive complaints and indicators of labour participation of older employees may be further advanced by taking into account factors from the work context. It is valuable to study the impact of work-related factors in this relation as this might reveal facilitating and/or hindering factors for labour participation of older employees with depressive complaints and as such give insight how the work context can be optimized for workers with depressive complaints. According to our theoretical framework, other available resources such as employment conditions may counteract individual reactions to stress (Grandey and Cropanzano 1999). This is also supported by previous empirical research. A favorable work context consisting amongst others of low physical work demands, experiencing decision latitude (Stynen et al. 2017), or support from supervisor(s) and co-worker(s) (Ilmarinen 2008) could buffer the adverse impact of depressive complaints. Moreover, employees are less likely to report having early retirement intentions (Stynen et al. 2017) and are more likely to report good work ability (Ilmarinen 2008). Contrary, an unfavorable work context consisting amongst others of high physical work demands and stressful work demands might elevate employees’ risk of being out of employment (Gommans et al. 2016; Lund et al. 2001). Moreover, employees are likely to report limited work ability (Ilmarinen 2008). These studies have shown that high psychological job demands, low decision latitude, high emotional demands, number of working hours and overtime work, are related with (an elevated risk of) depressive symptoms (Andrea et al. 2004; Bonde 2008; Driesen et al. 2010; Niedhammer et al. 2004). However, these studies often assessed work-related factors as risk factors for the occurrence of depressive complaints. To date little is known about the potential facilitating/hindering impact of the work context on the relation between depressive complaints and indicators of labour participation among older employees. The second goal of this study is therefore to acquire more in-depth insight into how older employees with depressive complaints could better facilitate their labour participation. This by assessing potential facilitating and/or hindering impact of factors from the work context such as physically demanding work, working hours, overtime work, decision latitude, psychological job demands, and emotional demands when studying the relation between depressive complaints and indicators of labour participation.

To conclude, our study aims to firstly assess associations between depressive complaints and indicators of labour participation among older employees cross-sectionally and longitudinally over a 2-year time period. Secondly, this study aims to assess the potential facilitating and/or hindering impact of factors from the work context in the relation between depressive complaints and indicators of labour participation.

## Materials and methods

### Maastricht cohort study

Data of the ongoing Maastricht Cohort Study (MCS) was used. The MCS is a large-scale prospective cohort study among employees in the Netherlands (Kant et al. 2003). At the baseline of this cohort (1998) 12,140 employees originating from 45 different companies/organizations were surveyed (Kant et al. 2003). Respondents received nine questionnaires in the period 1998 to 2002 with three additional follow-up questionnaires in 2008, 2012, and 2014. Details about the sampling, response rate, study population and measurements were reported elsewhere (Kant et al. 2003). For this study, the 2012 wave is considered as article baseline since the concepts depressive complaints and work ability being firstly addressed together in the same questionnaire. Data from the subsequent questionnaire in 2014 is used as 2-year follow-up wave for this study.

### Study population

At article baseline (2012), 4783 employees completed and returned the questionnaire. Firstly, employees younger than 45 years (*n* = 387) and at baseline not working or not implying being solely ‘actively employed’ (indicating being on (partial) sick leave or pregnancy leave) and not being employed by an employer or not responded to one of these questions (*n* = 2010) were excluded. Since the focus of this study is on workers employed by an employer, (partially) self-employed people (*n* = 60) were excluded. Also employees holding multiple jobs were excluded (*n* = 90) because of lacking information on the content of the second job. We excluded employees involved in shift work, night work or irregular working hours (*n* = 437) since particularly shift workers often suffer from disturbance of the circadian rhythm and social disruption, which have been linked to depressive complaints as well (Driesen et al. 2011). Additionally, shift work is associated with other labour participation outcomes (Gommans et al. 2015). Also employees with (pre-) pension arrangement(s) or occupational/sector specific pension arrangement(s) (*n* = 513) were excluded since our study population should still be actively employed at article baseline to assess incident cases of employees who lose (active) employment over time. Also employees on long-term sick leave (> 4 months) at article baseline (*n* = 19) were excluded. Furthermore, employees who did not complete all items required for calculating the baseline sum score of depressive complaints were excluded (*n* = 14). After applying these selection criteria, the total study population for the cross-sectional analyses consisted of *n* = 1253 employees (373 women and 880 men). For the longitudinal analyses prevalent cases of the respective outcomes were excluded to study incident cases only, resulting in *n* = 823 (good general work ability), *n* = 1171 (good physical work ability), *n* = 1,095 (good mental work ability), *n* = 1002 (weak retirement intentions), *n* = 1078 (low psychological disengagement), and *n* = 1253 (being in employment) still participating at the follow-up wave in 2014.

## Measurements

### Depressive complaints

The depression subscale (HAD-D) of the hospital anxiety and depression (HAD) scale was used to measure the presence and severity of depressive complaints (Zigmond and Snaith 1983). The HAD-D consists of 7 integrated items scored on a four-point Likert scale (0–3) resulting in a range of 0–21 (Lexis et al. 2009). As mentioned earlier, employees who did not complete all 7 items of the HAD-D scale at article baseline were excluded from the analyses as in this case no total HAD-D sum score could be calculated. A high value on the HAD-D indicates more severe depressive complaints (Lexis et al. 2009). Additionally, Zigmond and Snaith (1983) defined three categories/ranges of depressive complaints: a score of < 8 points was defined as a having no depressive complaints; a score of 8–10 points was defined as a having mild depressive complaints; and a score of 11 points of more was defined as having moderate/severe depressive complaints (Bjelland et al. 2002; Zigmond and Snaith 1983). In this study, the category ‘no depressive complaints’ is used as a reference group. Although the HAD-D scale was initially developed to identify (possible and probable) caseness of depression among patients in a nonpsychiatric hospital setting (Bjelland et al. 2002), the questionnaire has the same properties when being applied to other populations such as the general population (Bjelland et al. 2002; Lexis et al. 2009). The Cronbach’s alpha for internal consistency was 0.84 for the HAD-D reflecting a good internal consistency (Bjelland et al. 2002).

### Indicators of labour participation

#### Work ability

Work ability was operationalized by different items from the validated work ability index (WAI) (Ilmarinen 2007).

#### General work ability

General work ability was assessed by one item of the WAI—referred to as the work ability score (WAS). The WAS measures the self-assessed current work ability compared to lifetime best work ability on a ten-point scale varying from 0 (both physically and mentally unable to perform work) to 10 (better than ever able to work) (Ilmarinen 2007; Jääskeläinen et al. 2016). In line with previous studies (Boschman et al. 2017; Jääskeläinen et al. 2016), the WAS score was dichotomized: employees were considered having ‘poor to moderate work ability’ when scoring 0–7, and having ‘good work ability’ when scoring 8–10 (Boschman et al. 2017).

#### Mental and physical work ability

Two separate items of the WAI assessed mental and physical work ability by giving an overall rating on a 5-point scale (1 = ‘very bad’, 2 = ‘bad’, 3 = ‘moderate’, 4 = ‘good’, and 5 = ‘very good’). In line with earlier studies (Boschman et al. 2017; De Raeve et al. 2007; Ilmarinen 2008), the self-reported mental and physical work ability were dichotomized into ‘poor work ability’ (very bad, bad, moderate) and ‘good work ability’ (good, very good).

#### Retirement intentions

In line with other studies (Härkonmaki et al. 2006; Stynen et al. 2017), a single-item measured whether employees considered retiring before reaching mandatory retirement age: ‘have you considered retiring before reaching your mandatory retirement age?’ Response options ‘no’ and ‘yes, sometimes’ were defined as having weak retirement intentions and ‘yes, often’ as strong retirement intentions.

#### Motivation (psychological disengagement)

Psychological disengagement was assessed by six items regarding a variety of work investments, activities, and motivation—as an example ‘I think I should assign new responsibilities to younger persons’—which are expected to gradually decrease in older employees’ preretirement period (Damman et al. 2013). Answer options ranged from ‘completely agree’ (score 1) to ‘completely disagree’ (score 5) with a total score ranging from 5 to 30. A higher mean scale score represents higher psychological disengagement levels (Damman et al. 2013). The mean continuous scale score in this study was 12.69 (SD = 4.17) with an acceptable internal consistency of 0.71. Previous studies have not yet defined valid cut-off points to classify low and/or high disengagement levels. Within this study, the cut-off value was put on the ‘neutral score’ on the total scale (that is an average score on all six items being minimal ‘neutral’ (score 3)]. As such, employees with a mean scale score below 18.00 were regarded as having ‘low’ psychological disengagement levels, whereas employees with a mean scale score of 18.00 or higher were regarded as having ‘high’ psychological disengagement levels.

#### Work status

At article baseline, all employees reported being in current (paid) employment by an employer (inclusion criteria). At follow-up measurement, employees were again asked whether or not they were currently involved in paid employment (yes/no) by an employer (yes/no). Those reporting still being in paid employment were considered as ‘being in employment’. All other employees were classified as ‘not being in employment’, regardless of the underlying reason(s).

### Contextual and confounding factors

Based on previous research, potential confounding variables from demography/private situation, work context, and health domains, were identified (De Wind et al. 2014; Lee et al. 2017; Scharn et al. 2017). Confounding variables in this present study were measured at article baseline (2012).

#### Personal and health characteristics

Demographic variables comprised age, gender and educational level. Employees were asked to indicate their highest completed level of education—which was recoded into three categories: low (primary, lower vocational school); medium (lower secondary school, intermediate vocational school, upper secondary school); or high (higher vocational school, university) (Gommans et al. 2017). Household situation was assessed by employees indicating whether or not they were living alone (yes/no).

#### Work-related factors

This study identified potential confounding/contextual factors from the work context: psychological job demands, decision latitude, co-worker social support, supervisor social support, emotional demands, working hours, and physically demanding work. The validated Dutch version of the job content questionnaire (JCQ) was used to measure psychological job demands and decision latitude (Gründemann 1993). Psychological job demands were assessed by the sum of five items (such as excessive work) whereas decision latitude was measured by the sum of two subscales: skill discretion (such as job variety) and decision authority (such as having the freedom to make decisions). The total scores for the subscale psychological job demands ranges from 12 to 48 and for the subscale decision latitude ranges from 24 to 96. Two scales, each comprising four items, from the JCQ were used to measure co-worker social support and supervisor social support (total score range 4–16). All subscales from the JCQ had answer options on a four-point scale ranging from ‘strongly disagree’ to ‘strongly agree’. Emotional demands were measured by the sum of five items (such as being often confronted with the situation personally affecting employees), originating from the Dutch Questionnaire on the Experience and Evaluation of Work (VBBA) (Van Veldhoven and Broersen 2003; Van Veldhoven and Meijman 1994), the Dutch questionnaire on Work and Health (Gründemann 1993), and self-formulated. The total scale score ranges from 0 to 5. For the stratified analyses, the total subscale scores for psychological job demands, decision latitude, and emotional demands were calculated and split into tertiles resulting in low, middle and high scores. Based on these tertile scores, psychological job demands and emotional demands were grouped into low/middle and high levels, and decision latitude was grouped into low and middle/high levels. Working hours per week were assessed by one single item with five response options: > 40, 36–40, 26–35, 16–25, or < 16 h/week. These were recoded into full-time work (> 40 or 36–40 h/week) and part-time work (26–35, 16–25 or < 16 h/week). Employees were asked whether or not they often performed overtime work (yes/no). Whether or not employees considered their work physically demanding was assessed by one dichotomous item (yes/no) from the Dutch questionnaire on Work and Health (Gründemann 1993).

#### Presence of other mental/physical chronic condition(s)

The validated health and work performance questionnaire (HPQ) was used to identify the presence of 34 pre-specified health conditions (Kessler et al. 2003). To define the group of employees with chronic conditions and employees without chronic conditions (reference group), the 34 pre-specified conditions of the HPQ were matched with the ICPC-2 codes of the 28 pre-specified conditions listed by the Dutch National Institute of Public Health and the Environment (RIVM) (O’halloran et al. 2004; Van Oostrom et al. 2017). Several pre-specified health conditions on the HPQ list can be considered as determinants of disease(s), such as overweight, obesity or hypertension, and were thus not included in the listing of the RIVM as chronic condition (Sociaal-Economische Raad 2016). Seventeen chronic mental or physical conditions in the HPQ matched directly with the RIVM list. Depression was not included in the list of other mental/physical chronic conditions due to being the primary condition of interest within this study. This resulted in a total of 16 chronic mental or physical conditions on the HPQ list. Employees could indicate whether or not they have (a) chronic condition(s) and whether they had received or currently receive treatment for the health condition (Kessler et al. 2003). These answers were dichotomized into ‘no I don’t have’ or ‘yes, I have’. Employees were classified as having one or more other mental/physical chronic condition(s) when answering ‘yes, I have’ to one (or more) of these 16 chronic conditions. Furthermore, the HPQ-list ends with one open-ended question in which employees could list another chronic condition than listed above. The answers to this open-ended question were also matched with the ICPC-2 codes of the 28 chronic condition(s) as specified by the RIVM list. If a condition matched, employees were identified as having a chronic condition.

### Statistical analyses

All analyses were undertaken using SPSS Statistics IBM 22.0. *P* values below 0.05 were considered statistically significant. Differences at article baseline across the different categories of depressive complaints were examined by using one-way ANOVA for continuous measures and χ^2^ test of independence for categorical variables. For the cross-sectional analyses, logistic regression analyses were performed to examine the associations between depressive complaints both continuous and categorical and for the outcomes: general, mental, and physical work ability, retirement intentions, and psychological disengagement. Odds ratio’s (OR) and 95% confidence intervals (95% CI) were calculated. Three models were examined: in the first model adjustments were made for age, gender, educational level and living situation. In the second model, additional adjustments were made for psychological job demands, decision latitude, co-worker social support, supervisor social support, emotional demands, working hours, and physically demanding work. In the third model, additional adjustments were made for the presence of other mental/physical chronic condition(s). For the longitudinal analyses, Cox regression analyses were performed for all four outcome measurements. Prevalent cases for the respective indicator of labour participation at baseline were excluded to study only incident cases at follow-up. Hazard ratio’s (HR) and 95% CI were calculated. Here, the same adjustments were made step-wise as for the cross-sectional analyses. Moreover, stratified analyses were performed for possible facilitating and/or hindering factors from the work context. Depressive complaints were only assessed as a continuum in the stratified analyses. Again, prevalent cases for the respective indicator of labour participation were excluded and the same adjustments were made step-wise in line with the cross-sectional and longitudinal analyses.

## Results

### Descriptive results

The characteristics of the study population (*n* = 1253) and separately for the three categories of depressive complaints at 2012 baseline are reported in Table 1. Statistically significant differences were observed between the categories of depressive complaints regarding demographic and private factors (educational level and living alone). As for the work-related factors, statistically significant differences in mean scores for psychological job demands, decision latitude, co-worker social support, supervisor social support, emotional demands, and physically demanding work were observed between the different categories of depressive complaints. No statistically significant differences were observed for working hours. As for health factors, the percentage of employees having one or more mental/physical chronic condition(s) was significantly higher for employees with moderate/severe depressive complaints compared to employees without depressive complaints (85.3% vs 48.0). As for the indicators of labour participation, all outcome measures showed statistically significant differences in mean scores between the different categories of depressive complaints. At article baseline, a strong cross-sectional association between the severity of depressive complaints and retirement intentions was observed. Over 18 per cent of employees without depressive complaints indicated having strong retirement intentions compared to nearly 30% of employees with moderate/severe depressive complaints.

**Table 1:** Description of and mean depressive complaints scores of the study population (*n* = 1253) at article baseline (2012) according to demographic—and private, work and health factors (%)

	Total study population (*n* = 1253)	No depressive complaints ^(1)^ (*n* = 1144)	Mild depressive complaints ^(2)^ (*n* = 75)	Moderate/severe depressive complaints^(3)^ (*n* = 34)	*p* value
Demographic and private factors					
Age (years) Mean (SD)	54.60 (4.95)	54.60 (4.92)	54.28 (5.46)	55.15 (5.01)	0.695
Sex					
(%)Male (%)Female	70.2 29.8	70.0 30.0	69.3 30.7	79.4 20.6	0.491
Educational level					
(%) Low (%)Medium (%) High	5.3 23.0 71.7	4.9 23.2 71.9	4.1 21.6 74.3	21.9 18.8 59.4	0.001*
Living alone					
(% Yes)	10.6	9.8	22.7	9.1	0.002*
Health factors					
HAD-D score (mean, SD)\presence other chronic condition(s)	2.78 (3.08)	2.08 (2.05)	8.91 (.79)	12.85 (2.22)	< 0.0001*
(% Yes)	50.3	48.0	68.1	85.3	< 0.0001*
Work-related factors					
Working hours					
(%)Part-time (%) Full-time	29.8 70.2	29.8 70.2	32.4 67.6	24.2 75.8	0.694
Physically demanding work (% Yes)	13.7	12.8	18.6	32.4	0.002*
Mean (SD)					
Psychological job demands^a^	31.02 (5.64)	30.85 (5.54)	32.51 (6.29)	33.56 (6.38)	0.001*
Decision latitude^b^	75.00 (10.30)	75.42 (10.19)	71.73 (9.59)	68.00 (12.08)	< 0.0001*
Emotional demands^c^	1.05 (1.24)	0.99 (1.20)	1.44 (1.45)	2.06 (1.46)	< 0.0001*
Co-worker social support^d^	12.10 (1.51)	12.15 (1.49)	11.88 (1.53)	10.81 (1.53)	< 0.0001*
Supervisor social support^d^	10.95 (2.19)	11.03 (2.14)	10.27 (2.51)	9.76 (2.56)	< 0.0001*
Indicators of labour participation					
Work ability					
Mean (SD) General^e^ Physical^f^ Mental^f^	7.71 (1.17) 4.20 (.55) 4.04 (.57)	7.83 (1.05) 4.23 (.53) 4.10 (.52)	6.48 (1.63) 3.93 (.70) 3.54 (.67)	6.53 (1.44) 3.79 (.69) 3.29 (.63)	< 0.0001* < 0.0001* < 0.0001*
Retirement intentions (% Strong)	19.8	18.8	30.7	29.4	0.02*
Psychological disengagement Mean (SD)	12.70 (4.17)	12.43 (4.07)	15.51 (3.98)	15.35 (4.90)	< 0.0001*

(Andrea et al. 2004) No depressive complaints (HAD score ≤ 8); (Berendsen 2017) mild depressive complaints (HAD score = 8–10); (Bjelland et al. 2002) moderate/severe depressive complaints (HAD score ≥ 11)

^a^Scale range = 12–48

^b^Scale range = 24–96

^c^Scale range = 1–5

^d^Scale range = 4–16

^e^Scale range = 0–10

^f^Scale range = 0–5

### Cross-sectional and longitudinal associations between depressive complaints and indicators of labour participation

Cross-sectional and longitudinal associations between depressive complaints and indicators of labour participation at article baseline and over a two-year follow-up period are presented in respectively Tables 2 and 3. Only fully adjusted models are discussed. For the longitudinal analyses, prevalent cases for the respective indicator of labour participation at baseline were excluded.

**Table 2:** Cross-sectional associations between depressive complaints scores/categories and indicators of labour participation

	Poor general work ability			Poor physical work ability			Poor mental work ability			Strong retirement intentions			High psychological disengagement		
	OR^1^ (95% CI)	OR^2^ (95% CI)	OR^3^ (95% CI)	OR^1^ (95% CI)	OR^2^ (95% CI)	OR^3^ (95% CI)	OR^1^ (95% CI)	OR^2^ (95% CI)	OR^3^ (95% CI)	OR^1^ (95% CI)	OR^2^ (95% CI)	OR^3^ (95% CI)	OR^1^ (95% CI)	OR^2^ (95% CI)	OR^3^ (95% CI)
HAD-D continuous (0–21)	1.30 (1.24–1.37)*	1.26 (1.20–1.33)*	1.26 (1.20–1.33)*	1.30 (1.21–1.39)*	1.27 (1.17–1.37)*	1.24 (1.15–1.35)*	1.45 (1.37–1.55)*	1.41 (1.32–1.51)*	1.40 (1.31–1.50)*	1.10 (1.05–1.15)*	1.09 (1.04–1.14)*	1.09 (1.03–1.14)*	1.21 (1.14–1.27)*	1.14 (1.07–1.21)*	1.13 (1.06–1.20)*
Depressive complaints															
No^a^	1	1	1	1	1	1	1	1	1	1	1	1	1	1	1
Mild	6.69 (3.69–12.11)*	5.67 (3.08–10.47)*	5.54 (3.00–10.23)*	5.96 (2.86–12.42)*	5.22 (2.40–11.35)*	4.55 (2.07–9.97)*	6.15 (3.50–10.81)*	4.86 (2.69–8.77)*	4.57 (2.52–8.29)*	2.21 (1.27–3.86)*	2.00 (1.14–3.52)*	1.97 (1.12–3.47) *	2.85 (1.47–5.49)*	2.09 (1.04–4.20)*	1.95 (0.97–3.94)
Moderate/severe	5.93 (2.57–13.69)*	3.76 (1.56–9.06)*	3.57 (1.47–8.64)*	7.92 (3.11–20.15)*	4.90 (1.73–13.83)*	3.82 (1.35–10.87)*	17.38 (7.85–38.45)*	9.49 (3.98–22.60)*	8.11 (3.36–19.56)*	1.52 (0.66–3.52)	1.23 (0.51–2.95)	1.19 (0.49–2.87)	4.38 (1.87–10.26)*	2.21 (0.87–5.57)	1.91 (0.76–4.84)

^a^Reference group (Andrea et al. 2004) = adjusted for demographic and private factors (age, gender, educational level, living situation) (Berendsen 2017) = additionally adjusted for work-related factors (working hours, psychological job demands, decision latitude, emotional demands, co-worker social support, supervisor social support and physical demands) (Bjelland et al. 2002) = additionally adjusted for the presence of other mental/physical chronic condition(s)

**Table 3:** Longitudinal associations between depressive complaints and indicators of labour participation over 2-year follow-up period

	Poor general work ability			Poor physical work ability			Poor mental work ability			Strong retirement intentions			High psychological disengagement			Not being in employment		
	HR^1^ (95% CI)	HR^2^ (95% CI)	HR^3^ (95% CI)	HR^1^ (95% CI)	HR^2^ (95% CI)	HR^3^ (95% CI)	HR^1^ (95% CI)	HR^2^ (95% CI)	HR^3^ (95% CI)	HR^1^ (95% CI)	HR^2^ (95% CI)	HR^3^ (95% CI)	HR^1^ (95% CI)	HR^2^ (95% CI)	HR^3^ (95% CI)	HR^1^ (95% CI)	HR^2^ (95% CI)	HR^3^ (95% CI)
HAD-D continuous (0–21)	1.09 (1.03–1.16)*	1.08 (1.00–1.15)*	1.07 (1.00–1.15)*	1.09 (1.00–1.19)*	1.08 (0.99–1.19)	1.08 (0.98–1.18)	1.26 (1.18–1.35)*	1.22 (1.13–1.31)*	1.21 (1.12–1.31)*	1.00 (0.94–1.07)	0.96 (0.89–1.03)	0.96 (0.89–1.03)	1.08 (1.02–1.14)*	1.07 (1.01–1.14)*	1.07 (1.01–1.13)*	1.03 (1.00–1.07)	1.03 (0.99–1.06)	1.02 (0.99–1.06)
Depressive complaints																		
No^a^	1	1	1	1	1	1	1	1	1	1	1	1	1	1	1	1	1	1
Mild	1.91 (0.78–4.70)	1.67 (0.66–4.23)	1.66 (0.66–4.19)	2.49 (0.87–7.09)	2.26 (0.77–6.61)	2.22 (0.76–6.50)	3.94 (1.84–8.43)*	2.85 (1.28–6.37)*	2.60 (1.14–5.92)*	1.24 (0.54–2.86)	0.97 (0.41–2.28)	0.97 (0.41–2.28)	2.52 (1.32–4.81)*	2.38 (1.22–4.62)*	2.35 (1.21–4.57)*	1.33 (0.87–2.03)	1.28 (0.83–1.96)	1.26 (0.82–1.94)
Moderate/severe	2.23 (0.70–7.12)	1.92 (0.56–6.59)	1.79 (0.52–6.20)	2.03 (0.46–8.93)	1.58 (0.34–7.20)	1.53 (0.33–6.98)	8.76 (3.00–25.58)*	4.66 (1.43–15.21)*	4.22 (1.27–14.00)*	0.78 (0.19–3.20)	0.50 (0.12–2.17)	0.50 (0.12–2.16)	1.03 (0.25–4.21)	0.94 (0.22–3.95]	0.91 (0.22–3.86]	1.23 (0.67–2.26]	1.09 (0.58–2.06]	1.07 (0.56–2.03]

^a^Reference group (Andrea et al. 2004) = adjusted for demographic and private factors (age, gender, educational level, living situation) (Berendsen 2017) = additionally adjusted for work-related factors (working hours, psychological job demands, decision latitude, emotional demands, co-worker social support, supervisor social support and physical demands) (Bjelland et al. 2002) = additionally adjusted for the presence of other mental/physical chronic condition(s)

### General, mental and physical work ability

At article baseline, a one-point increase in HAD-D score was statistically significantly associated with higher odds for poor general, physical, and mental work ability (respectively OR 1.26, 95% CI 1.20–1.33; OR 1.24, 95% CI 1.15–1.35; and OR 1.40, 95% CI 1.31–1.50) after controlling for confounding factors. Employees with mild and moderate/severe depressive complaints had statistically significantly higher odds for poor general work ability (respectively OR 5.54, 95% CI 3.00–10.23; and OR 3.57, 95% CI 1.47–8.64) and poor physical work ability (respectively OR 4.55, 95% CI 2.07–9.97; and OR 3.82, 95% CI 1.35–10.87) compared to employees without depressive complaints. Furthermore, employees with mild and moderate/severe depressive complaints had statistically significantly higher odds for poor mental work ability (OR 4.57, 95% CI 2.52–8.29; and OR 8.11, 95% CI 3.36–19.56) compared to employees without depressive complaints. At follow-up measurement, a one-point increase in HAD-D was borderline statistically significantly associated with a higher risk for poor general work ability (HR 1.07, 95% CI 1.00–1.15], after controlling for confounding factors. Moreover, a one-point increase in HAD-D was substantially and statistically significantly associated with a higher risk for poor mental work ability (HR 1.21, 95% CI 1.12–1.31). Employees with mild and moderate/severe depressive complaints had substantial and statistical significant higher risks for poor mental work ability (respectively HR 2.60, 95% CI 1.14–5.92; and HR 4.22, 95% CI 1.27–14.00) over time compared to employees without depressive complaints. No statistically significantly associations were found for poor physical work ability.

### Retirement intentions

At article baseline, a one-point increase in HAD-D score was statistically significantly associated with higher odds for strong retirement intentions (OR 1.09, 95% CI 1.03–1.14). Employees with mild depressive complaints had higher odds for strong retirement intentions (OR 1.97, 95% CI 1.12–3.47) compared to employees without depressive complaints. At follow-up measurement, a one-point increase in HAD-D was not statistically significantly associated with a higher risk for strong retirement intentions. Either, employees with mild and moderate/severe depressive complaints had no statistical significant higher risk for strong retirement intentions over time, compared to employees without depressive complaints.

### Psychological disengagement

At article baseline, a one-point increase in HAD-D was statistically significantly associated with higher odds for high psychological disengagement levels (OR 1.13, 95% CI 1.06–1.20). At follow-up measurement, a one-point increase in HAD-D was statistically significantly associated with a higher risk for high psychological disengagement levels (HR 1.07, 95% CI 1.01–1.13). Employees with mild depressive complaints had a substantial and statistical significant higher risk for high psychological disengagement levels (HR 2.35, 95% CI 1.21–4.57) over time compared to employees without depressive complaints. However, no statistical significant associations were found for employees with moderate/severe depressive complaints.

### Employment status

No cross-sectional associations were assessed since all employees were in (paid) employment at article baseline. When assessing the risk of not being in employment at follow-up measurement, fully adjusted models showed no statistical significant higher risk, while the first model (solely adjusted for demographic and private factors) showed that a one-point increase in HAD-D was borderline statistically significantly associated with a higher risk of not being in employment (HR 1.03, 95% CI 1.00–1.07) over time. No statistical significant associations were found for employees with mild and moderate/severe depressive complaints.

Association between depressive complaints and indicators of labour participation over-time: stratification for potentially hindering and/or facilitating work-related factors.

To explore the potential facilitating and/or hindering impact of work-related factors on the strength of the association between depressive complaints and indicators of labour participation over time, stratified analyses were performed for the following six factors: physically demanding work, working hours, overtime work, decision latitude, psychological job demands, and emotional demands (Table 4). Depressive complaints were only assessed as a continuum. Prevalent cases for the respective indicator of labour participation at baseline were excluded.

**Table 4:** Associations between depressive complaints and indicators of labour participation over 2-year follow-up period: stratification for potentially facilitating/hindering work-related factors

	Poor general work ability			Poor physical work ability			Poor mental work ability			Strong retirement intentions			High psychological disengagement			Not being in employment		
	HR^1^ (95% CI)	HR^2^ (95% CI)	HR^3^ (95% CI)	HR^1^ (95% CI)	HR^2^ (95% CI)	HR^3^ (95% CI)	HR^1^ (95% CI)	HR^2^ (95% CI)	HR^3^ (95% CI)	HR^1^ (95% CI)	HR^2^ (95% CI)	HR^3^ (95% CI)	HR^1^ (95% CI)	HR^2^ (95% CI)	HR^3^ (95% CI)	HR^1^ (95% CI)	HR^2^ (95% CI)	HR^3^ (95% CI)
HAD-D continuous (0–21)																		
Physically demanding work																		
Yes	1.12 (0.94–1.34)	1.07 (0.87–1.32)	1.09 (0.88–1.35)	1.17 (1.02–1.35)*	1.18 (0.99–1.41)	1.23 (1.01–1.50)*	1.23 (1.05–1.43)*	1.44 (1.06–1.96)*	1.44 (1.05–1.97)*	0.88 (0.73–1.07)	0.82 (0.67–1.00)	0.82 (0.67–1.01)	1.14 (0.99–1.30)*	1.15 (0.98–1.35)	1.14 (0.97–1.34)	1.03 (0.96–1.12)	1.04 (0.96–1.13)	1.04 (0.95–1.13)
No	1.08 (1.01–1.16)*	1.07 (0.99–1.16)	1.06 (0.99–1.15)	1.05 (0.93–1.17)	1.04 (0.92–1.18)	1.05 (0.93–1.19)	1.27 (1.17–1.37)*	1.21 (1.11–1.32)*	1.21 (1.10–1.32)*	1.02 (0.95–1.10)	0.99 (0.92–1.07)	0.99 (0.91–1.07)	1.07 (1.00–1.14)*	1.07 (1.00–1.14)*	1.07 (1.00–1.14)*	1.03 (0.99–1.07)	1.02 (0.98–1.06)	1.02 (0.98–1.06)
Working hours																		
Full-time	1.11 (1.04–1.19)*	1.08 (1.00–1.17)*	1.08 (1.00–1.16)*	1.14 (1.03–1.26)*	1.09 (0.97–1.22)	1.09 (0.97–1.22)	1.29 (1.20–1.39)*	1.22 (1.11–1.33)*	1.20 (1.09–1.32)*	1.00 (0.93–1.08)	0.96 (0.88–1.05)	0.95 (0.88–1.04)	1.06 (0.99–1.14)	1.05 (0.98–1.13)	1.05 (0.98–1.13)	1.02 (0.98–1.07)	1.01 (0.97–1.06)	1.01 (0.97–1.06)
Part-time	1.00 (0.84–1.20)	1.02 (0.85–1.24)	1.02 (0.84–1.23)	0.98 (0.81–1.19)	1.00 (0.82–1.23)	1.00 (0.81–1.22)	1.18 (1.01–1.37)	1.25 (1.04–1.50)*	1.26 (1.04–1.52)*	0.99 (0.87–1.13)	0.94 (0.81–1.09)	0.95 (0.82–1.11)	1.17 (1.04–1.31)*	1.22 (1.06–1.40)*	1.21 (1.06–1.39)*	1.05 (0.99–1.11)	1.05 (0.99–1.12)	1.05 (0.99–1.13)
Overtime work																		
Yes	1.10 (1.00–1.21)*	–	–	1.09 (0.92–1.29)	–	–	1.30 (1.17–1.45)*	–	–	0.99 (0.88–1.11)	–	–	1.10 (0.99–1.22)*	–	–	1.02 (0.95–1.08)	1.01 (0.94–1.08)	1.00 (0.93–1.08)
No	1.08 (1.00–1.18)*	1.07 (0.98–1.18)	1.06 (0.97–1.16)	1.08 (0.98–1.19)	1.09 (0.98–1.21)	1.09 (0.98–1.21)	1.25 (1.15–1.37)*	1.25 (1.13–1.39)*	1.24 (1.11–1.38)*	1.01 (0.93–1.09)	0.99 (0.91–1.08)	0.99 (0.90–1.08)	1.06 (0.99–1.14)	1.06 (0.99–1.14)	1.06 (0.99–1.14)	1.04 (1.00–1.08)*	1.03 (0.99–1.08)	1.04 (0.99–1.08)
Decision latitude																		
Low	1.05 (0.96–1.14)	1.05 (0.95–1.15)	1.04 (0.95–1.14)	1.10 (0.99–1.23)	1.09 (0.96–1.24)	1.09 (0.96–1.24)	1.31 (1.19–1.45)*	1.28 (1.15–1.42)*	1.27 (1.14–1.41)*	0.98 (0.90–1.06)	0.96 (0.88–1.05)	0.96 (0.87–1.05)	1.06 (0.98–1.15)	1.05 (0.97–1.14)	1.05 (0.96–1.14)	1.04 (1.00–1.08)*	1.03 (0.99–1.08)	1.04 (0.99–1.08)
Middle/high	1.13 (1.03–1.23)*	1.13 (1.02–1.25)*	1.13 (1.02–1.25)*	1.12 (0.96–1.31)	1.10 (0.94–1.29)	1.09 (0.93–1.29)	1.20 (1.07–1.34)*	1.16 (1.02–1.32)*	1.17 (1.02–1.34)*	1.01 (0.91–1.13)	0.99 (0.88–1.12)	0.99 (0.88–1.12)	1.10 (1.01–1.20)*	1.14 (1.05–1.25)*	1.15 (1.05–1.25)*	1.04 (1.00–1.08)*	1.03 (0.99–1.08)	1.04 (0.99–1.08)
Psychological job demand																		
High	1.13 (1.02–1.25)*	1.10 (0.99–1.24)	1.10 (0.98–1.24)	1.19 (1.02–1.38)*	1.15 (0.97–1.37)	1.14 (0.95–1.36)	1.24 (1.11–1.38)*	1.16 (1.03–1.33)*	1.15 (1.01–1.32)*	0.97 (0.86–1.08)	0.88 (0.77–1.00)	0.86 (0.75–.99)	1.13 (1.00–1.29)*	1.08 (0.92–1.26)	1.07 (0.91–1.24)	0.98 (0.90–1.06)	0.96 (0.88–1.05)	0.96 (0.87–1.05)
Low/middle	1.06 (0.98–1.16)	1.06 (0.96–1.16)	1.06 (0.96–1.16)	1.03 (0.91–1.17)	1.05 (0.92–1.20)	1.05 (0.92–1.20)	1.25 (1.15–1.37)*	1.28 (1.16–1.42)*	1.29 (1.16–1.44)*	1.01 (0.93–1.10)	0.99 (0.91–1.08)	1.00 (0.91–1.09)	1.07 (1.01–1.14)*	1.06 (1.00–1.14)*	1.06 (1.00–1.14)*	1.05 (1.01–1.08)*	1.03 (0.99–1.07)	1.03 (0.99–1.07)
Emotional demands																		
High	1.08 (1.01–1.17)*	1.08 (0.99–1.17)	1.08 (0.99–1.17)	1.09 (0.99–1.19)	1.10 (0.99–1.22)	1.09 (0.98–1.21)	1.22 (1.13–1.32)*	1.23 (1.13–1.33)*	1.23 (1.13–1.33)*	1.01 (0.94–1.09)	0.99 (0.91–1.08)	1.00 (0.91–1.09)	1.09 (1.02–1.16)*	1.07 (1.00–1.15)*	1.08 (1.00–1.16)*	1.02 (0.98–1.07)	1.02 (0.98–1.07)	1.02 (0.98–1.07)
Low/middle	1.11 (0.98–1.26)	1.06 (0.93–1.21)	1.06 (0.92–1.21)	1.07 (0.86–1.34)	1.08 (0.85–1.36)	1.07 (0.85–1.36)	1.31 (1.11–1.56)*	–	–	0.96 (0.84–1.11)	.91 (0.79–1.06)	0.91 (0.78–1.05)	1.06 (0.95–1.19)	1.05 (0.94–1.18)	1.05 (0.94–1.18)	1.03 (0.97–1.09)	1.02 (0.96–1.08)	1.02 (0.96–1.08)

Andrea et al. 2004) = adjusted for demographic and private factors (age, gender, educational level, living situation) (Berendsen 2017) = additionally adjusted for work-related factors (working hours, psychological job demands, decision latitude, emotional demands, and physical demands) (Bjelland et al. 2002) = additionally adjusted for the presence of other mental/physical chronic condition(s)

### Physically demanding work

Among employees reporting to perform physically demanding work, results showed substantial and statistical significant associations between depressive complaints and poor physical (HR 1.23, 95% CI 1.01–1.50) and mental (HR 1.44, 95% IC 1.05–1.97) work ability over time. No statistical significant associations were found with between depressive complaints and other indicators of labour participation. Among employees reporting not to perform physically demanding work, a substantial and statistical significant association was found between depressive complaints and poor mental work ability (HR 1.21, 95% CI 1.10–1.32) over time, and a borderline significant association was found between depressive complaints and high psychological disengagement levels (HR 1.07, 95% CI 1.00–1.14) over time.

### Working hours

Among full-time workers, results showed a borderline statistically significant association between depressive complaints and poor general work ability (HR 1.08, 95% CI 1.00–1.16) over time. Also, among full-time workers, results showed a substantial and significant association between depressive complaints and mental work ability (HR 1.20, 95% CI 1.09–1.32) over time. Among part-time workers, results showed a statistically significant association between depressive complaints and poor mental work ability (HR 1.26, 95% CI 1.04–1.52) and a substantial and statistically significant association between depressive complaints and high psychological disengagement levels (HR 1.21, 95% CI 1.06–1.39) over time.

### Overtime work

Among employees reporting to work overtime, no statistically significant results were obtained. Among employees reporting not to work overtime, results showed a significant association between depressive complaints and poor mental work ability (HR 1.24, 1.11–1.38) over time.

### Decision latitude

Among employees reporting low decision latitude, results showed a substantial and statistically significant association between depressive complaints and poor mental work ability (HR 1.27, 95% CI 1.14–1.41) over time. No statistically significant associations between depressive complaints and other indicators of labour participation were observed. Among employees reporting middle/high decision latitude, results showed a statistically significant association between depressive complaints and poor general work ability (HR 1.13, 95% CI 1.02–1.25) and high disengagement levels (HR 1.15, 95% CI 1.05–1.25) over time.

### Psychological job demands

Among employees reporting high psychological job demands, results showed a borderline statistically significant association between depressive complaints and poor mental work ability (HR 1.15, 95% CI 1.01–1.32) over time. No statistical significant associations were found between depressive complaints and poor general and physical work ability. Among employees reporting low/middle psychological job demands, results showed a substantial and statistical significant high risk for poor mental work ability (HR 1.29, 95% CI 1.16–1.44) over time.

### Emotional demands

Among employees reporting high emotional demands, results showed a borderline statistical significant association between depressive complaints and high psychological disengagement levels (HR 1.08, 95% CI 1.00–1.16) over time. Also, among employees reporting high emotional demands, results showed a substantial association between depressive complaints and poor mental work ability (HR 1.23, 95% CI 1.13–1.33) over time. No statistical significant associations between depressive complaints and other indicators of labour participation were observed. Among employees reporting low/middle emotional demands, results showed no statistically significant associations between depressive complaints and indicators of labour participation over time.

## Discussion

The aim of this prospective study was to examine associations between depressive complaints and indicators of labour participation among older employees and to explore the possible facilitating and/or hindering impact of several work-related factors from the work context on this relation.

Cross-sectional results show that depressive complaints as a continuum were significantly associated with elevated odds of poor general, physical, mental work ability, strong retirement intentions, and high psychological disengagement levels among older employees even after controlling for confounding factors. Employees with mild and moderate/severe depressive complaints had substantial and significantly higher odds for poor general, physical, and mental work ability compared to employees without depressive complaints. In addition, employees with mild depressive complaints had significantly higher odds for strong retirement intentions compared to employees without depressive complaints.

Longitudinal results show that depressive complaints as a continuum were associated with a higher risk of reporting poor general and mental work ability as well as higher psychological disengagement levels over time. Employees with mild and moderate/severe depressive complaints had a substantial statistical significant higher risk for poor mental work ability over time compared to employees without depressive complaints. The observed results are in line with the COR theory. Employees with mild and moderate/severe depressive complaints might have or perceive a loss of resources in terms of health. This loss of resources affects employees rapidly and thus might lead to negative outcomes such as withdrawal or disengagement from work activities (Hobfoll 2018) affecting labour participation. Additionally, the COR theory explains that ageing is accompanied with an inevitable loss of resources. This may explain the strong associations observed in this study since our study population consists of older employees. Previous studies also support the strong associations observed between depressive complaints and high psychological disengagement levels. Declining health might accelerate the work disengagement process (Damman et al. 2013; Ilmarinen 2008). Additionally, results indicate substantial associations between depressive complaints and poor mental work ability over time. This might be explained by depressive complaints being considered a potential precursor for depression. Previous research already showed the negative affect of clinical depression on labour participation outcomes such as a decreased work ability and productivity loss (Ilmarinen 2008; Lagerveld et al. 2010). Despite being a precursor for depression, depressive complaints thus also appear to substantially negatively affect labour participation outcomes. No statistically significant associations were found between depressive complaints and strong retirement intentions and not being in employment over time.

Analyses furthermore explored the potential facilitating and/or hindering role of factors from the work context on the relation between depressive complaints and indicators of labour participation. The observed results show that the work context plays an important role in (the strength of) the adverse association between depressive complaints and indicators of labour participation over time among employees reporting to perform physically demanding work or having high psychological job demands. Results show that older employees reporting to perform physically demanding work, having high psychological job demands, having low decision latitude, or having high emotional demands, were at higher risk to report having poor mental work ability over time. These findings are supported by previous studies which also state that diseases, in general, have a detrimental effect on work ability. These detrimental effects are most apparent among workers performing physically demanding work (Ilmarinen 2008). Previous research also shows that high job demands negatively affect employees’ physical and mental health (Karasek 1979; Stansfeld et al. 2013). Furthermore, the observed results show that employees reporting having high emotional demands were at higher risk to report high psychological disengagement levels over time. According to the COR theory ageing is accompanied by an inevitable loss of resources such as impaired health (Hobfoll 2018). However, various (individual) resources might minimize these losses due to variations in self-esteem or skills (Grandey and Cropanzano 1999). Perhaps employees with high emotional demands in our study do at the same time have sufficient job resources such as more skills or a higher self-esteem. Consequently, these employees are thus not that affected by the loss of resources such as health. These employees might be able to cope with depressive complaints in their working life. Among part-time workers, a substantial-high risk was found between depressive complaints and high psychological disengagement levels over time. The strength of this association was lower and not significant among full-time workers. Perhaps employees previously involved in full-time employment yet experiencing (mild) depressive complaints reduced their working hours to part-time employment before article baseline to remain (actively) involved in paid employment. We cannot rule out a potential healthy-worker effect due to various selection processes. The impact of these (potential) selection processes on the interpretation of our study results will be explained more in-depth in the internal validity paragraph.

### Internal validity

The internal validity of this study is strengthened amongst others due to the use of the validated HAD-D scale (Zigmond and Snaith 1983). Although the HAD-D was initially designed to detect depressive complaints in a nonpsychiatric hospital setting, the validity of the HAD-D scale is also warranted in other settings such as primary care patients, in the general population (Bjelland et al. 2002) and among employees (Andrea et al. 2004). The validity of the HAD-D among employees is additionally substantiated by the distribution of employees indicating to be currently under treatment by doctor/caregiver for depressive complaints at article baseline, which increases with increasing severity of depressive complaints (specific data not presented). The HAD-D scale allowed to measure depressive complaints both as a continuum and categorical. This provided more in-depth insights within different categories of severity of depressive complaints. Also, the continuous HAD-D score could indicate a transition and/or increase in indicators per one-point increase in HAD-D. Another major advantage of this study is the prospective and large cohort design. This prospective design enables us to assess cause and effect and to study longitudinal associations between depressive complaints and indicators of labour participation over time while taking into account confounding factors. Despite this longitudinal design, various selection processes and healthy worker effects (Neophhytou et al. 2014) cannot be ruled out. At article baseline, a potential healthy worker effect might have occurred due to selective participation. Perhaps employees experiencing (more severe) depressive complaints might have already left the labour force or have adjusted their work situation by reducing the amount of working hours and/or (perhaps) enrolled in part-time work due to their health problems already earlier. This might also explain the relatively small proportion of employees with moderate/severe depressive complaints (*n* = 34). Unfortunately, information about the duration of (a) depressive (episode) complaints is lacking at article baseline. We should interpret these results carefully since this might be a selected population of employees being still actively employed despite having severe depressive complaints. Results are thus likely to be an underestimation of the true impact of depressive complaints on these indicators of labour participation. Moreover, mean HAD-D score at article baseline was statistically significantly higher for the non-responders (*n* = 174) compared to the responders (*n* = 1,079) at follow-up measurement (specific data not shown). This selective loss to follow-up over time may have attenuated the results regarding the impact of depressive complaints on indicators of labour participation over time. Moreover, in both the cross-sectional and longitudinal analyses, several adjustments were made for important demographic, work-related, and health factors. These confounding variables could potentially bias or modify the relation between depressive complaints and indicators of labour participation. In general, ORs and HRs moderately decreased after an additional adjustment was made. After additionally controlling for the presence of other mental/physical chronic condition(s) HRs showed a small decrease. The presence of one or more other chronic condition(s) was highly prevalent among the study population and especially among employees with moderate/severe depressive complaints (85.3%). Perhaps controlling for the presence of other chronic condition(s) led to an underestimation of the true strength of the associations. In the longitudinal analyses, the strength of the association substantially decreased after additionally controlling for work-related factors. This illustrated a potential impact from the work context when studying this relation. Moreover, the observed differences between cross-sectional and longitudinal findings might be (partially) attributable to the duration and frequency of the follow-up period.

### External validity

Within our study, we aimed to complement the concept labour participation by assessing both objective and subjective outcome measures. The generalizability in our study is strongly affected by the chosen outcome measure. For instance, the generalizability of the observed results in terms of employment status and retirement intentions may be restricted to employees in the Netherlands since these outcomes are also determined by external factors such as social security systems. However, (general, physical, and mental) work ability and psychological (dis)engagement are universal outcome measures which are not country-specific. These observed results are thus readily generalizable to other European countries. In contrast, having (strong) retirement intentions is highly affected and influenced by the social insurance systems within a specific country. This makes the observed results with regards to strong retirement intentions less generalizable to other settings.

### Recommendations further research

Employees without depressive complaints were classified as reference group in which results showed substantial and significant differences compared to, respectively, employees with mild depressive complaints and employees with moderate/severe depressive complaints. For further research, it might be interesting to test differences between employees with mild depressive complaints and moderate/severe depressive complaints. By exploring a (potential) difference in the severity of depressive complaints, preventive measures might be tailored specifically for the severity of depressive complaints.

Furthermore, within this study various objective and subjective indicators were used to complement the concept labour participation. This is specifically valuable when studying older employees since (declining) health status not only affects work ability but might also affect retirement intentions. A more comprehensive understanding is obtained which might contribute to developing tailored preventive measures and thus optimize labour participation of older employees. However, it should be acknowledged that this study was not intended to be exhaustive in examining all (possible) indicators of labour participation. Further research might for instance explore indicators of labour participation in further detail by assessing the underlying reasons for not being in employment. The exit route from paid employment through for instance disability schemes or unemployment benefits could be explored. While this study explored the potential role of six work-related factors from the work context when studying the relation between depressive complaints and indicators of labour participation, it should be noted that the stratified analyses were based on single work-related factors from the work context and thus not exhaustive. Further research should aim to gain more in-depth insight into the role of other work-related factor(s) from the work context, such as task variety or competencies when studying this relation.

Despite the longitudinal study design being a major prerequisite to assess a causal association over time between depressive complaints and indicators of labour participation, no measurements have taken place in the meantime. Further research should consider more frequent measurements during follow-up to detect more cases of (mild) depressive complaints.

Our goal was to assess whether there is an association between depressive complaints and indicators of labour participation among older employees over time. To increase this study internal validity a more homogenous group of older employees was assembled. Results show substantial results amongst these employees involved in daywork. However, it is also valuable to assess this association in other settings. Therefore, further research should investigate this association in other groups of older employees working for instance irregular working hours or being involved in night and/or shift work.

### Concluding remarks

Overall, this study indicates strong, adverse associations between increasing depressive complaints and indicators of labour participation over time. Moreover, different work contexts revealed various negative associations between depressive complaints and indicators of labour participation. However, these results were not unambiguous since we found differential effects indicating the important role of the work context when studying this relation.

To prevent the transition from depressive complaints to clinical depression, work organizations/employers should aim to monitor depressive complaints among employees. Uniform preventive measures and/or interventions for all older employees are difficult since a one-size fits-all measure is probably ineffective considering the impact of different work contexts. In line with the COR theory, preventive measures or interventions might focus on enhancing resources to compensate for the losses in other resources such as health (Hobfoll 2018). Work organizations/employers should focus on balancing job demands and job resources (such as more autonomy) since this could have a beneficial impact on labour participation indicators among older employees. In addition, it is valuable to consider the role of the work context since the strength of these associations substantially differs within various work contexts. In practice, these study findings may serve as valuable input when considering the development of preventive measures and/or interventions aiming to prolong older employees’ working life.

In sum, further research on the relation between depressive complaints and indicators of labour participation should further explore the underlying reason(s) of (early) labour market exit. In addition, more in-depth insights into other factors from the work context is necessary when studying this relation, to optimize labour participation of older employees in a sustainable way.
